# Machine Learning with Multiparametric MRI and Clinical Biomarkers for Noninvasive Renal Interstitial Fibrosis Staging

**DOI:** 10.3390/bioengineering13060704

**Published:** 2026-06-19

**Authors:** Kexin Wang, Tao Zhao, Tao Su, Yizhu Jiang, Lei Jiang, Jianxing Qiu, Shuo Quan, Jiangtao Liu, Rui Wang

**Affiliations:** 1 Department of Radiology, Peking University First Hospital, Beijing 100034, China; kexin_wang@pku.edu.cn (K.W.); qjx761225@126.com (J.Q.); 15201504354@163.com (S.Q.); 2 Renal Division, Peking University First Hospital, Peking University Institute of Nephrology, Beijing 100034, China; zhaot0713@bjmu.edu.cn (T.Z.); tao.su@bjmu.edu.cn (T.S.); jiangyizhu2001@163.com (Y.J.); jianglei@bjmu.edu.cn (L.J.); 3 Beijing Smart Tree Medical Technology Co., Ltd., Beijing 102200, China; liujiangtao@smart-imaging.cn

**Keywords:** kidney, fibrosis, machine learning, multiparametric magnetic resonance imaging

## Abstract

Renal interstitial fibrosis (RIF) is currently assessed by invasive biopsy. This prospective study developed and validated a noninvasive random forest model combining multiparametric MRI and clinical biomarkers for identifying severe RIF in 116 patients with biopsy-confirmed renal disease. Quantitative parameters were extracted from IVIM, ASL, phase-contrast MRI, T1 mapping, and BOLD sequences. Fibrosis was classified as mild (<25%) or severe (≥25%). In the held-out test set, the random forest model achieved an AUC of 0.89 (95% CI 0.82–0.96), sensitivity of 0.91, and specificity of 0.73, significantly outperforming clinical-only (AUC 0.63), MRI-only (AUC 0.63), and combined LASSO logistic regression (AUC 0.73) benchmarks. The model also demonstrated superior calibration (Brier score 0.154) and net clinical benefit on decision curve analysis. This integrated MRI–clinical model shows promise for noninvasive identification of severe RIF and warrants external prospective validation.

## 1. Introduction

Renal interstitial fibrosis (RIF) constitutes a critical pathological endpoint in chronic kidney disease, serving as the final common pathway to end-stage renal disease [1]. The severity of RIF directly correlates with progression risk to end-stage renal disease, rendering accurate histopathological staging essential for prognostication, therapeutic decision-making, and treatment response monitoring [2]. Percutaneous renal biopsy remains the current reference standard for RIF assessment, but carries inherent risks, including bleeding complications, sampling variability, and procedural contraindications exist for high-risk patients with coagulopathies or solitary kidneys [3]. Conventional noninvasive biomarkers, such as serum creatinine, estimated glomerular filtration rate, and proteinuria, demonstrate insufficient sensitivity and specificity for precise fibrosis staging, particularly in early disease stages where functional compensation may mask underlying structural damage [4,5].

Multiparametric MRI has emerged as a promising noninvasive alternative for evaluating renal parenchymal diseases [6,7,8]. Functional MRI techniques probe diverse aspects of renal pathophysiology: intravoxel incoherent motion (IVIM) quantifies tissue cellularity and microstructural disruption through separation of perfusion and diffusion components [4,9]; T1 mapping provides insights into tissue composition and free-water content, reflecting interstitial expansion [10]; blood oxygen level-dependent (BOLD) MRI assesses tissue oxygenation and hypoxia as key drivers of fibrogenesis [11]; arterial spin labeling (ASL) measures renal perfusion; and phase-contrast MRI provides renal artery flow hemodynamic information without exogenous contrast [7,12,13]. Renal pathophysiology is complex, with early-stage compensatory hyperfiltration and vascular adaptations contrasting with advanced-stage decompensation. Consequently, reliance on any single MRI parameter proves insufficient to capture RIF’s multifaceted progression, often yielding ambiguous interpretations and limiting diagnostic accuracy.

Artificial intelligence’s integration with multiparametric MRI represents a promising solution to these limitations by capturing nonlinear relationships across pathophysiological domains. While machine learning applications in renal texture analysis show potential, no existing study has integrated clinical biomarkers with comprehensive multiparametric functional MRI for RIF staging across both acute and chronic kidney disease spectra. This gap is particularly significant given that acute kidney injury significantly increases the risk for the development of chronic kidney disease, necessitating diagnostic approaches applicable across disease states.

We hypothesize that an artificial intelligence model synthesizing clinical biomarkers and multiparametric MRI can accurately classify RIF severity by transcending the limitations of single-parameter analysis. This study aimed to develop a machine learning framework integrating multiparametric MRI and clinical biomarkers for noninvasive RIF staging.

## 2. Materials and Methods

### 2.1. Patients

This prospective study was conducted at two centers within a single institution and approved by the Institutional Review Board (IRB No. 2022-503, 2023-039), with written informed consent obtained from all participants.

**Figure 1:**
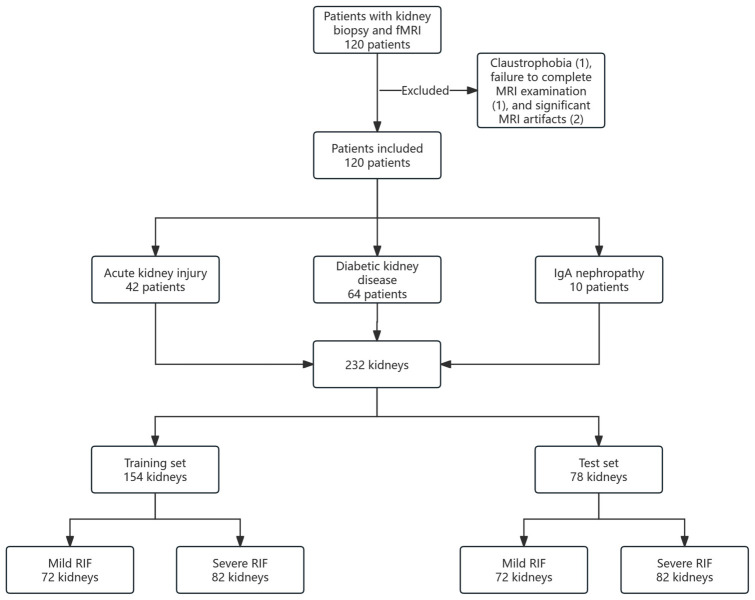
Flowchart of the study cohort recruitment and dataset allocation.

Between August 2022 and April 2025, 120 consecutive patients with biopsy-confirmed renal disease were initially enrolled from two centers (Figure 1). Eligible disease categories included acute kidney injury due to acute tubulointerstitial nephritis, and chronic kidney disease due to diabetic kidney disease or IgA nephropathy. The inclusion criteria were: (1) age ≥15 years; (2) histopathological diagnosis confirmed by percutaneous renal biopsy; and (3) absence of concomitant renal diseases unrelated to the primary diagnosis. The exclusion criteria were: (1) claustrophobia or inability to tolerate MRI; (2) failure to complete the full MRI examination protocol; and (3) significant MRI image artifacts precluding quantitative analysis, including motion artifacts or image degradation due to extensive ascites. Four patients were subsequently excluded due to claustrophobia (*n* = 1), incomplete MRI examination (*n* = 1), and significant MRI artifacts (*n* = 2), resulting in a final cohort of 116 patients.

### 2.2. MRI Acquisition

All participants underwent renal multiparametric MRI within one week post-biopsy using 3.0 T scanners (Ingenia, Philips Healthcare, Best, the Netherlands) with a 32-channel body array coil. Standardized noncontrast protocols included: axial Dixon T1-weighted imaging, coronal T2-weighted imaging, IVIM with seven b-values (0, 10, 20, 50, 100, 500, 1000 s/mm^2^), T1 mapping with seven inversion times (350, 550, 750, 950, 1150, 1350, 1650 ms), three-dimensional pseudo-continuous ASL, BOLD with 12 echoes, and phase-contrast MRI with two-directional velocity encoding set to 150 cm/s for bilateral renal artery flow quantification. Detailed acquisition parameters are provided in Table S1. The total scan time was 19 min 32 s including patient positioning, localizer scans, sequence preparations, and acquisition times.

### 2.3. Image Analysis

Image processing was performed independently by two blinded readers: a nephrologist with specialized training in renal imaging and four years of experience, and an abdominal radiologist with 14 years of expertise in genitourinary imaging. Kidney segmentation was manually performed, generating total kidney volume and maximal coronal kidney length on coronal T2-weighted imaging. The height-adjusted total kidney volume was calculated as total kidney volume divided by height (m). The quantitative parameters were measured on the following maps: pure diffusion coefficient (D), perfusion fraction (f), and pseudo-diffusion coefficient (D*) maps derived from IVIM; a renal blood flow (RBF) map derived from ASL; a T1 map derived from T1 mapping; a T2* map derived from BOLD; and flux, stroke volume, and velocity values derived from phase-contrast MRI.

On coronal renal hilum sections, regions of interest (ROIs) were manually placed at the upper, middle, and lower poles, avoiding biopsy tracts, the collecting system, vessels, and artifacts. For each kidney, one cortical ROI (5–15 cm^2^, tracing the outer renal contour) and five medullary ROIs (15–30 mm^2^ each) were placed (Figure S1).

Cortico-medullary differences (denoted as Δ) were calculated as cortical minus medullary values. Cortico-medullary boundary integrity was graded on T1 maps using a pre-defined three-point scale: 1 = distinct boundary, 2 = blurred boundary, 3 = obliterated boundary. Independent reader measurements were compared systematically, with discrepancies resolved through consensus review with a third senior radiologist.

### 2.4. Clinical and Histopathological Assessment

Clinical biomarkers were extracted from electronic health records, including demographic information, body mass index, laboratory values of hemoglobin, albumin, serum creatinine, estimated glomerular filtration rate, mean arterial pressure, C-reactive protein, D-dimer, blood urea nitrogen (BUN), and 24 h urine total protein (UTP). Histopathological evaluation was initially reviewed by one nephropathologist with 10 years of experience and subsequently validated by a senior nephropathologist with 25 years of experience. Fibrosis severity was quantitatively assessed, with mild RIF defined as involvement of <25% of the area affected, and severe RIF defined as involvement of ≥25%.

### 2.5. Data Analysis

Before analysis, the entire cohort was randomly divided at the patient level into a training set (70%, *n* = 77 patients, 154 kidneys) and an internal held-out test set (30%, *n* = 39 patients, 78 kidneys) using stratified sampling to ensure balanced distribution of RIF severity (mild vs. severe) and major disease categories. All clinical biomarkers and multiparametric MRI parameters from both kidneys of each patient were kept together within the same set. This patient-level splitting strategy eliminated data leakage from paired kidneys or shared clinical variables (e.g., UTP and BUN).

The analysis proceeded in three main stages. First, pairwise correlations and group comparisons were performed to explore relationships among variables and identify differences between mild and severe RIF groups. Second, the predictive value of individual parameters was assessed using univariable logistic regression. Third, multivariable models were developed, including penalized logistic regression (LASSO) models and a random forest model, to capture both linear and nonlinear relationships.

All feature selection, hyperparameter tuning, and model optimization were conducted exclusively within the training set. The final models were locked and evaluated on the internal held-out test set.

### 2.6. Random Forest Model Development and Internal Validation

A nested resampling strategy was used to develop and internally validate the random forest model while minimizing the risk of overfitting.

Data splitting: The dataset was divided at the patient level into a training cohort and an independent test cohort. The independent test cohort was withheld from all feature selection, hyperparameter tuning, and model development procedures and was used exclusively for final model evaluation.

Internal resampling procedure: Within the training cohort, repeated stratified 5-fold cross-validation was performed using the caret package in R (version 4.4.1). Cross-validation was repeated 10 times to improve the stability of model performance estimates. Stratification was performed according to RIF severity (mild vs. severe) to preserve class balance across folds.

Feature selection: Feature selection was conducted using recursive feature elimination (RFE) implemented in the caret package. The RFE procedure was performed within the repeated stratified 5-fold cross-validation framework (repeated 10 times) using random forest functions. Model performance was evaluated using the area under the receiver operating characteristic curve (AUC). Feature subsets of varying sizes were iteratively evaluated, and the optimal subset of seven variables was selected based on the highest mean cross-validated AUC. In addition, we assessed the stability of feature selection by examining the frequency with which each variable was retained across the repeated cross-validation folds.

Hyperparameter tuning: Hyperparameter optimization was conducted within the resampling framework using grid search implemented through the train() function in caret. The following tuning grid was evaluated: ntree: 300, 500, 800; maxnodes: 8, 10, 12, unrestricted; nodesize: 1, 2, 4. In addition, the number of candidate variables randomly sampled at each split (mtry) was optimized automatically within the cross-validation procedure. A fixed random seed (set.seed(123)) was used throughout to ensure reproducibility.

Out-of-bag and internal validation performance: In addition to repeated cross-validation, out-of-bag (OOB) estimates generated by the random forest algorithm were used as an additional internal validation measure. Model discrimination was assessed using area under the curve (AUC) values derived from OOB predictions for the repeated cross-validation. OOB error convergence analysis was performed to evaluate model stability during training. OOB error rates for the overall model and for each class were monitored as the number of trees increased.

Final model construction and evaluation: The optimal feature subset and hyperparameter combination were selected according to the mean cross-validated AUC within the training cohort. The final random forest model was subsequently retrained using the entire training cohort and evaluated on the independent held-out test cohort.

Software: All statistical analyses and machine learning procedures were performed in R software (version 4.3.1) using the caret, randomForest, and pROC packages.

Variable importance was evaluated using both mean decrease in Gini impurity and permutation-based importance (mean decrease in accuracy). Permutation importance was included to reduce the potential bias associated with Gini importance and to provide a more robust model-agnostic assessment of predictor contribution.

To further evaluate model learning dynamics and potential optimism during random forest construction, training set AUC and OOB AUC were calculated at regular intervals (every 50 trees) as the number of trees increased from 50 to 500. These convergence curves were used to visualize the evolution of model discrimination performance and the gap between training and internal validation AUC.

### 2.7. Development of LASSO Logistic Regression Models for Ablation Study

To evaluate the incremental value of multiparametric MRI features and to provide a fair statistical benchmark, LASSO-penalized logistic regression models were developed using the identical patient-level training and test cohorts as the random forest model.

Three model variants were constructed with the same feature sets: clinical variables only, MRI variables only, and combined (clinical + MRI variables).

LASSO logistic regression was implemented using the glmnet package in R. The regularization parameter (λ) was optimized via repeated stratified 5-fold cross-validation (repeated 10 times) within the training cohort, using AUC as the performance metric. The optimal λ was selected based on the highest mean cross-validated AUC, with the 1-SE rule also considered for parsimony. A fixed random seed (set.seed(123)) was used for reproducibility. Final models were refitted on the entire training cohort and evaluated on the held-out test cohort.

### 2.8. Diagnostic Performance Evaluation

The diagnostic performance of the final random forest model was compared with the three LASSO logistic regression models (clinical-only, MRI-only, and combined) and with individual significant parameters. Performance was assessed on the independent internal test set using receiver operating characteristic (ROC) analysis, reporting the area under the curve (AUC) with 95% confidence intervals, sensitivity, specificity, positive predictive value (PPV), and negative predictive value (NPV).

Calibration was evaluated using calibration plots, slope, intercept, and Brier score. Clinical utility was assessed by decision curve analysis (DCA). The DeLong test was used to compare AUC values between models. All analyses were performed in R software (version 4.3.1).

### 2.9. Statistical Analysis

Statistical analyses were performed in R (version 4.3.1). Continuous variables are reported as medians with interquartile ranges or means ± standard deviations according to data distribution, and were compared between groups using the Mann–Whitney U test. Categorical variables are summarized as frequencies and percentages, and were compared using the chi-square test or Fisher’s exact test as appropriate. Inter-observer reproducibility of quantitative MRI parameters was assessed using intraclass correlation coefficients, and cortico-medullary boundary grading reliability was assessed using weighted kappa. AUC values were compared between models using the DeLong test. Statistical significance was set at a two-tailed *p* < 0.05. *p*-values in Table 1 and Table 2 were adjusted for multiple comparisons using the false discovery rate (FDR) method, while *p*-values in Table 3 are presented as unadjusted values, as is conventional for exploratory association analyses.

## 3. Results

### 3.1. Patient Laboratory Characteristics, Demographics, and Pathology Findings

The study cohort comprised 116 patients (mean age 51.3 ± 13.6 years), including 72 men (mean age 51.2 ± 13.3 years) and 44 women (mean age 51.6 ± 13.9 years), with no significant age difference between different sexes (*p* = 0.84). Overall, 42 patients with acute tubulointerstitial nephritis, 64 with diabetic kidney disease, and 10 with IgA nephropathy with biopsy-confirmed pathology were recruited after exclusions for claustrophobia (*n* = 1), failure to complete MRI examination (*n* = 1), and significant MRI artifacts (*n* = 2). Patients were stratified by histopathology into mild RIF (<25% fibrosis, *n* = 51) and severe RIF (≥25% fibrosis, *n* = 65) groups. As detailed in Table 1, baseline characteristics showed no statistically significant differences in age, sex distribution, or body mass index between the two groups (all *p* > 0.05). However, the severe RIF group demonstrated a 2.24-fold increase in UTP (median: 3.37 g/24 h vs. 1.04 g/24 h; *p* < 0.001) compared with the mild RIF group. No significant differences were observed between the two groups in serum creatinine, estimated glomerular filtration rate, hemoglobin, albumin, C-reactive protein, D-dimer, BUN, or mean arterial pressure (all *p* > 0.05).

### 3.2. Multiparametric MRI Findings

Inter-reader reproducibility was robust for quantitative MRI parameters, with intraclass correlation coefficients ranging from 0.65 to 0.85 (Table S2). Cortico-medullary boundary grading demonstrated excellent inter-reader agreement (weighted κ = 0.88).

**Figure 2:**
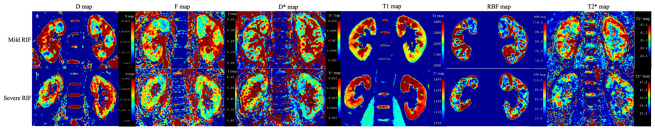
Representative multiparametric MRI maps in mild versus severe renal interstitial fibrosis (RIF) groups. (**a**) Multiparametric maps in a 62-year-old woman with acute kidney injury secondary to biopsy-proven acute tubulointerstitial nephritis (mild RIF; histopathologic fibrosis extent <25%). (**b**) Multiparametric maps in a 44-year-old man with biopsy-confirmed diabetic kidney disease (severe RIF; histopathologic fibrosis extent ≥25%). From left to right: intravoxel incoherent motion (IVIM)-derived pure diffusion coefficient (D), perfusion fraction (f), and pseudo-diffusion coefficient (D*) maps, T1 mapping-derived T1 map, arterial spin labeling (ASL)-derived renal blood flow (RBF) map, and blood oxygen level-dependent (BOLD)-derived T2* map.

MRI characteristics stratified by RIF severity are detailed in Table 2 and Figure 2. Morphometric parameters, including kidney length (*p* = 0.81) and height-adjusted total kidney volume (*p* = 0.31), did not differ significantly between groups. Cortical D was significantly lower in the severe RIF group compared with the mild RIF group (1.35 [1.28, 1.43] × 10^−3^ mm^2^/s vs. 1.44 [1.31, 1.54] × 10^−3^ mm^2^/s; *p* < 0.001). Similarly, ΔD was 1.50-fold lower in the severe RIF group (*p* < 0.001), indicating disruption of tubular microarchitecture and loss of diffusion anisotropy. Cortico-medullary boundary obliteration was more frequent in the severe RIF group than in the mild RIF group (70/130 kidneys [53.8%] vs. 20/102 kidneys [19.6%]; *p* < 0.001). ΔT1 was 32.9% less negative in the severe RIF group compared with the mild RIF group (−108 ms vs. −161 ms; *p* < 0.001), reflecting interstitial expansion, collagen deposition, and altered free-water distribution. Regarding renal hemodynamics, renal artery flux (−10.5%; *p* = 0.01), stroke volume (−16.6%; *p* = 0.01), velocity (−22.5%; *p* < 0.001), and cortical renal blood flow (−14.4%; *p* = 0.004) were all significantly lower in the severe RIF group. Cortical and medullary T2* values did not differ significantly between groups (*p* = 0.90 and *p* = 0.11, respectively), suggesting preserved baseline oxygenation despite structural and hemodynamic alterations.

### 3.3. Correlation and Regression Analyses

Pairwise correlation analysis revealed a strong positive association between renal artery flux and stroke volume (r = 0.92), indicating tightly coupled vascular flow dynamics. All other variable pairs demonstrated low-to-moderate correlations (all r < 0.80).

Univariable logistic regression (Table 3) identified UTP (odds ratio [OR]: 2.72, per 1-SD = 4.65 g/24 h increase; 95% CI: 1.62–4.46, *p* < 0.001), pathology disease diagnosis (IgA nephropathy OR: 6.60, 95% CI: 1.22–35.60, *p* = 0.03; diabetic kidney disease OR: 4.74, 95% CI: 2.34–9.58, *p* < 0.001), and ten other MRI parameters as significant predictors of severe RIF group (all *p* < 0.05). In multivariable analysis adjusting for potential confounders, nine independent predictors retained significance: hemoglobin (OR: 2.50, *p* = 0.005), BUN (OR: 1.99, *p* = 0.04), UTP (OR: 2.72, *p* = 0.02), ΔD (OR: 1.00, *p* = 0.004), medullary D* (OR: 0.36, *p* = 0.008), cortical T1 (OR: 2.51, *p* = 0.02), stroke volume (OR: 0.22, *p* = 0.02), velocity (OR: 0.38, *p* = 0.01), and medullary T2* (OR: 0.45, *p* = 0.006).

### 3.4. Random Forest Model Development and Performance

**Figure 3:**
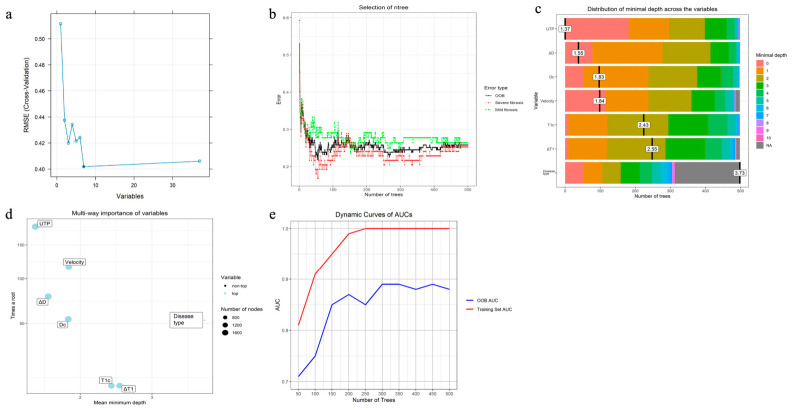
Development of the random forest model for renal interstitial fibrosis (RIF) classification. (**a**) Recursive feature elimination process. The plot shows that the optimal feature size was seven, achieving the lowest root-mean-square error (root-mean-square error [RMSE]: 0.45). (**b**) Out-of-bag (OOB) error rate and class-specific error rates as a function of the number of trees (ntree) during random forest model development. The overall OOB error and class-specific error rates decreased rapidly during the early stages of forest growth and stabilized after approximately 150–200 trees, indicating convergence of the random forest model with increasing ensemble size. The selected ntree value of 500 was therefore considered sufficient to achieve stable error estimates. Complementary training set and OOB AUC convergence analyses are presented in Figure 3e. (**c**) Distribution of minimal depth across variables in the random forest model. (**d**) Multi-way importance plot of variables in the random forest model. (**e**) Dynamic changes in training set and OOB AUC during random forest construction. AUC values were calculated at regular intervals as the number of trees increased from 50 to 500.

The recursive feature elimination process was employed to optimize the model by systematically reducing the number of features based on their importance. The progression of the root-mean-square error from cross-validation was tracked throughout this process, as depicted in Figure 3a. The optimal number of features was determined to be seven, at which point the model achieved its lowest root-mean-square error of 0.45. These seven features were then used for the development of a random forest model. Cross-validation was used to tune the model and optimize its performance by finding the best number of trees, which was determined to be 500 (Figure 3b), and the best number of variables to consider at each split in the decision trees, which was found to be four.

As shown in Figure 3b, the OOB error rapidly decreased during the early training phase and subsequently plateaued as the number of trees increased, indicating stable model convergence without progressive performance deterioration. The overall OOB error stabilized after approximately 150–200 trees, supporting the adequacy of the selected random forest configuration.

The importance of variables for the random forest model in predicting RIF is presented in Table 4 and Figure 3c,d. The major predictors identified by Gini importance, including UTP, ΔD, cortical D, velocity, and T1-related parameters, also demonstrated high permutation importance (mean decrease in accuracy), supporting the robustness of these imaging biomarkers in model discrimination (Table 4).

As shown in Figure 3e, the training set AUC increased progressively from 0.81 at 50 trees to 1.00 after approximately 250 trees and remained stable thereafter. In contrast, the OOB AUC increased rapidly during the early stages of forest growth, reaching approximately 0.89 at 300 trees and subsequently plateauing. Although a performance gap between training and OOB AUC was observed, the gap remained relatively stable after convergence and did not continue to widen with increasing ensemble size. These findings suggest moderate model optimism but do not support progressive overfitting during forest growth.

### 3.5. Ablation Study Using LASSO Logistic Regression Models

**Figure 4:**
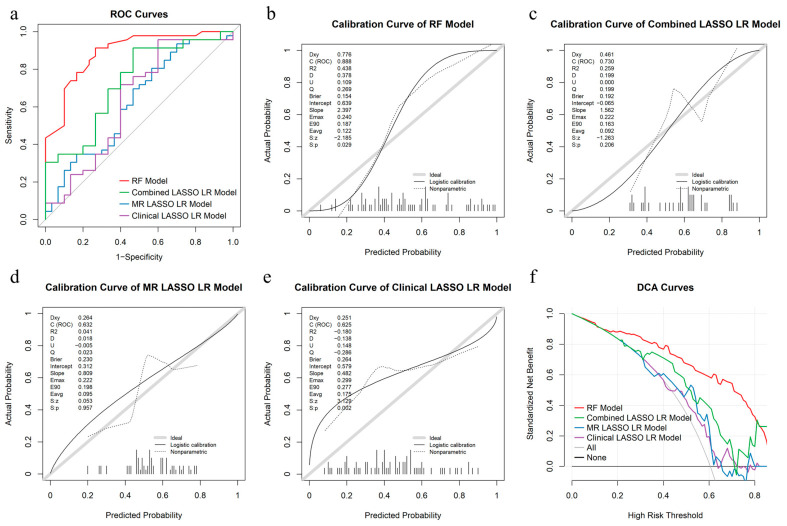
Receiver operating characteristic (ROC), calibration, and decision curve analysis (DCA) curves in the internal held-out test set. (**a**) ROC curves of the random forest (RF) model and the three LASSO logistic regression (LR) models in the internal held-out test cohort. (**b**) Calibration curve of the RF model for predicting kidney fibrosis. (**c**) Calibration curve of the combined LASSO LR model. (**d**) Calibration curve of the MR LASSO LR model. (**e**) Calibration curve of the clinical LASSO LR model. (**f**) Decision curve analysis (DCA) of predictive models for kidney fibrosis.

The classification performance of the RF model and the three LASSO logistic regression models is summarized in Table 5. The ROC curves for the internal held-out test set are presented in Figure 4a. Among the LASSO-based models, the combined clinical + MRI model achieved the highest discriminative performance, with an AUC of 0.730 (95% CI: 0.613–0.848), outperforming both the MRI-only model (AUC: 0.632, 95% CI: 0.494–0.770) and the clinical-only model (AUC: 0.625, 95% CI: 0.493–0.758). These findings suggest that combining clinical and MRI-derived features improved predictive performance compared with either feature category alone.

However, the RF model consistently demonstrated superior performance compared with all LASSO logistic regression variants, achieving an AUC of 0.890 (95% CI: 0.816–0.964), an accuracy of 0.842, a sensitivity of 0.913, and a specificity of 0.733 in the independent test cohort. DeLong tests demonstrated statistically significant superiority of the RF model over the combined clinical + MRI model (*p* = 0.033), the MRI-only model (*p* = 0.003), and the clinical-only model (*p* < 0.001).

Calibration curves are shown for all four models (Figure 4b–e). The RF model achieved the highest discrimination (C-statistic = 0.888) with a Brier score of 0.154. The DCA curves are presented in Figure 4f. The RF model consistently demonstrated the highest net benefit across the widest range of threshold probabilities, followed by the combined LASSO LR model. 

## 4. Discussion

This study presents an integrated clinical-MRI machine learning framework for noninvasive RIF severity classification. Our main findings demonstrate that a multiparametric approach combining clinical biomarkers (UTP) and functional MRI parameters, particularly cortico-medullary pure diffusion coefficient (ΔD) and cortical T1, achieves favorable diagnostic accuracy (AUC 0.88) for distinguishing mild from severe RIF. This approach maintains robust performance across a spectrum of acute and chronic renal diseases.

UTP and ΔD emerged as top predictors with biological relevance. UTP is a well-established clinical biomarker in chronic kidney disease correlating with RIF progression [14]. However, its specificity for RIF is limited [15]. Variability due to non-fibrotic factors (e.g., hydration, diet, transient inflammation), and its weaker correlation with biopsy-confirmed RIF, hinder its standalone classification accuracy [15]. Conversely, functional MRI techniques provide direct, quantitative insights into tissue microstructure and perfusion, consistently showing reproducible correlations with histological fibrosis scores [10,11,16,17]. The 1.5-fold decrease in ΔD in severe fibrosis reflects loss of cortico-medullary diffusion anisotropy, pathologically explained by collagen deposition disrupting tubular architecture and water mobility [16,18]. Notably, cortical T1 elevation further corroborates extracellular matrix accumulation [10,19]. The 22.53% reduction in renal artery velocity and 14.41% decline in cortical blood flow suggests progressive vascular rarefaction—a hallmark of severe fibrosis [20]. This complementary nature, UTP indicating systemic biochemical dysfunction and functional MRI revealing localized tissue alterations, provides a compelling rationale for their integration. Our findings support this approach: UTP emerged as the most important predictor in our models. Crucially, integrating UTP with BUN and MRI parameters (especially ΔD and T1 values) showed higher diagnostic value than UTP alone, demonstrating synergistic value for accurate RIF severity classification.

While previous studies provided proof-of-concept for functional MRI in RIF, their cohorts were limited to chronic kidney disease and smaller sample sizes (*n*= 80 and 43, respectively) [21,22]. The present dual-center cohort (*n* = 116) incorporates the pathophysiological continuum from acute tubulointerstitial nephritis through diabetic kidney disease and IgA nephropathy, better reflecting real-world nephrology practice where fibrosis manifests heterogeneously across injury types [23,24].

Our multiparametric MRI protocol interrogates distinct pathophysiological domains: IVIM quantifies microstructural integrity, ASL and phase-contrast MRI assess macro- and microvascular hemodynamics, T1 mapping evaluates interstitial expansion, and BOLD MRI probes tissue oxygenation. This comprehensive profiling addresses the biological heterogeneity of fibrosis progression more effectively than single-parameter approaches.

The random forest model’s superior performance (AUC 0.88 vs. logistic regression’s 0.59 in internal held-out test set; *p* < 0.001) aligns with emerging evidence that ensemble methods can effectively integrate multidimensional data in renal pathology. Logistic regression assumes linear decision boundaries and often underfits complex patterns [25]. In contrast, random forest captures nonlinear relationships and feature interactions inherent in integrated clinical-MRI data, albeit at the cost of interpretability and computational intensity [26]. The performance gap observed in our study likely stems from random forest’s ability to model intricate interactions between clinical variables and functional MRI features that violate logistic regression’s linearity assumption.

Our study contributes to the field by developing an integrated machine learning framework that synthesizes clinical and multiparametric MRI data to generate predictive probabilities—a prerequisite for translating MRI into clinical practice. With a sensitivity of 0.90 and specificity of 0.73 against the histopathological reference standard, our noninvasive model shows potential to identify severe RIF. In clinical practice, this model could be integrated into a tiered workflow. Nephrologists could first assess readily available clinical biomarkers such as UTP and BUN. Patients with indeterminate risk could then undergo multiparametric MRI, with the model providing a probability-based recommendation to inform subsequent management. For example, a predicted probability above 0.70 may support stronger consideration of aggressive immunosuppressive therapy in IgA nephropathy or diabetic kidney disease, where fibrosis extent dictates treatment intensity, while a predicted probability below 0.30 may help avoid unnecessary biopsy in low-risk patients. This approach may prove particularly valuable in clinical scenarios where biopsy is contraindicated [27,28], or to inform the decision to proceed with biopsy in indeterminate cases, thereby reducing procedural risks. Nevertheless, biopsy remains the reference standard for RIF assessment, and the current evidence supports only the hypothesis that this integrated MRI–clinical model may assist in risk stratification. External validation and prospective studies are required before clinical implementation can be considered.

Our study has several limitations. First, our MRI protocol was optimized exclusively for a single-vendor scanner. Broader clinical adoption will require cross-vendor standardization to ensure reproducibility across diverse imaging platforms. Second, although manual ROI placement demonstrated moderate inter-reader agreement, this approach inherently carries observer variability. Developing automated segmentation pipelines would improve objectivity and scalability. Third, MRI was performed within one week after renal biopsy in all patients. Although biopsy tracts were avoided during ROI placement, the potential influence of post-biopsy changes (such as hematoma or local edema) on MRI-derived parameters cannot be fully excluded. Future studies should ideally perform MRI prior to biopsy to eliminate this potential confounder. Fourth, although the cohort included a spectrum of renal diseases (acute tubulointerstitial nephritis, diabetic kidney disease, and IgA nephropathy), these entities differ substantially in etiology and baseline characteristics. Disease type alone showed relatively good performance in the test set, suggesting that the model may partially capture disease-specific features in addition to fibrosis severity. Due to limited subgroup sizes (especially IgA nephropathy, *n* = 10), disease-stratified analyses were not feasible. Thus, generalizability across individual disease etiologies requires further validation in larger cohorts. Fifth, the model was validated on an independent internal held-out test set. Although the dual-center design provides some improvement in generalizability, external validation in independent cohorts with different scanner vendors and acquisition protocols is still necessary. Future large-scale, multi-etiology studies are warranted.

## 5. Conclusions

This study demonstrates that integrating clinical biomarkers and multiparametric MRI, particularly pure diffusion coefficient (ΔD) and tissue characterization (T1), via a random forest model is a feasible approach for the noninvasive classification of RIF severity. This internally validated approach holds potential promise for enhancing risk stratification, therapeutic monitoring, and clinical decision-making in renal diseases. Further research is needed to confirm the generalizability of our results in larger, multi-center cohorts, and further disease-specific validation is warranted.

## Figures and Tables

**Table 1:** Clinical characteristics of the study cohort.

Clinical Characteristics	Overall (*n* = 116)	Fibrosis Group			Disease Type			
		Mild RIF (*n* = 51)	Severe RIF (*n* = 65)	*p* Value	Acute Kidney Injury (*n* = 42)	Diabetic Kidney Disease (*n* = 64)	IgA Nephropathy (*n* = 10)	*p* Value
Age (y)	51.3 ± 13.6	53.7 ± 13.8	49.4 ± 13.2	0.10	55.2 ± 14.0	50.6 ± 12.5	39.3 ± 12.0	0.003
Sex				0.66				<0.001
Female	44 (37.9%)	21 (41.2%)	23 (35.4%)		26 (61.9%)	14 (21.9%)	4 (40.0%)	
Male	72 (62.1%)	30 (58.8%)	42 (64.6%)		16 (38.1%)	50 (78.1%)	6 (60.0%)	
Body mass index (kg/m^2)^	25.24 ± 4.0	24.88 ± 3.81	25.53 ± 4.15	0.39	23.52 [21.22, 25.23]	26.24 [24.03, 29.26]	25.51 [22.44, 26.54]	0.001
Mean arterial pressure (mmHg)	100.83 ± 13.23	99.37 ± 12.54	101.98 ± 13.73	0.29	96.62 ± 13.31	102.53 ± 12.88	107.70 ± 10.56	0.02
Serum creatinine (μmol/L)	311.07 ± 219.40	294.21 ± 239.88	324.29 ± 202.81	0.47	339.14 ± 248.40	285.60 ± 185.96	356.16 ± 285.23	0.38
Estimated glomerular filtration rate (mL/min/1.73 m^2^)	23.74 [12.65, 42.32]	25.31 [13.60, 45.80]	22.81 [12.16, 32.91]	0.14	22.60 [10.25, 30.74]	24.61 [13.54, 44.06]	23.74 [11.70, 37.65]	0.08
Hemoglobin (g/L)	111.0 ± 23.4	109.86 ± 22.59	111.91 ± 24.19	0.64	106.8 ± 23.8	112.1 ± 22.44	121.9 ± 26.1	0.16
Albumin (g/L)	36.82 ± 6.55	38.15 ± 6.34	35.77 ± 6.58	0.052	40.09 ± 6.24	34.71 ± 6.19	36.56 ± 4.58	<0.001
C-reactive protein (mg/L)	7.73 ± 15.23	7.72 ± 11.28	7.74 ± 17.81	0.99	3.23 [0.92, 14.39]	1.35 [0.65, 5.29]	2.93 [0.90, 4.92]	0.04
D-dimer (mg/L)	0.20 [0.09, 0.44]	0.15 [0.08, 0.45]	0.21 [0.11, 0.42]	0.65	0.24 [0.11, 0.66]	0.16 [0.08, 0.40]	0.13 [0.11, 0.40]	0.06
BUN (mmol/L)	14.04 ± 6.60	12.76 ± 6.77	15.04 ± 6.33	0.06	13.42 ± 7.20	14.66 ± 6.25	12.66 ± 6.29	0.51
UTP (g/24 h)	2.00 [0.57, 5.32]	1.04 [0.40, 2.38]	3.37 [1.00, 6.58]	<0.001	0.50 [0.32, 0.97]	4.10 [2.01, 8.01]	3.17 [1.46, 5.45]	<0.001

Note: Continuous variables are reported as medians with interquartile ranges or means ± standard deviations, depending on data distribution. Categorical variables are summarized as frequency counts (*n*) and percentages (%). *p*-values were adjusted for multiple comparisons using the false discovery rate (FDR) method. RIF; renal interstitial fibrosis; BUN: blood urea nitrogen; UTP: urine total protein.

**Table 2:** Multiparametric MRI characteristics of renal interstitial fibrosis (RIF) evaluation.

MRI Parameters	Overall (*n* = 232)	Fibrosis Severity			Acute Kidney Injury			Diabetic Kidney Disease			IgA Nephropathy		
		Mild RIF (*n* = 102)	Severe RIF (*n* = 130)	*p* Value	Mild RIF (*n* = 60)	Severe RIF (*n* = 24)	*p* Value	Mild RIF (*n* = 40)	Severe RIF (*n* = 88)	*p* Value	Mild RIF (*n* = 2)	Severe RIF (*n* = 19)	*p* Value
Kidney length (cm)	10.40 ± 1.14	10.40 ± 1.16	10.40 ± 1.13	0.81	10.20 ± 1.02	10.30 ± 1.02	0.76	10.60 ± 1.29	10.50 ± 1.20	0.64	8.96 ± 1.04	9.95 ± 0.78	0.40
Height-adjusted total kidney volume (mL/m)	72.40 [58.70, 91.60]	74.30 [61.60, 92.10]	72.00 [58.40, 91.00]	0.31	69.50 [58.70, 81.50]	65.70 [58.50, 84.60]	0.15	77.40 [66.50, 98.00]	73.90 [65.30, 96.70]	0.83	59.40 ± 4.88	57.40 ± 11.10	0.68
Cortico-medullary boundary				<0.001			0.40			<0.001			<0.001
Distinct boundary	14 (6.0%)	10 (9.8%)	4 (3.1%)		8 (13.3%)	2 (8.3%)		2 (5.0%)	2 (2.3%)		0 (0%)	0 (0%)	
Blurred boundary	128 (55.2%)	72 (70.6%)	56 (43.1%)		42 (70.0%)	15 (62.5%)		30 (75.0%)	35 (39.8%)		0 (0%)	6 (33.3%)	
Obliterated boundary	90 (38.8%)	20 (19.6%)	70 (53.8%)		10 (16.7%)	7 (29.2%)		8 (20.0%)	51 (58.0%)		2 (100%)	12 (66.7%)	
Dc (×10^−3^ mm^2^/s)	1.38 [1.29, 1.48]	1.44 [1.31, 1.54]	1.35 [1.28, 1.43]	<0.001	1.38 [1.28, 1.48]	1.35 [1.23, 1.41]	0.10	1.49 ± 0.10	1.36 ± 0.12	<0.001	1.40 ± 0.04	1.37 ± 0.12	0.52
Dm (×10^−3^ mm^2^/s)	1.38 ± 0.13	1.39 ± 0.13	1.38 ± 0.12	0.32	1.36 ± 0.13	1.37 ± 0.12	0.77	1.45 ± 0.10	1.38 ± 0.12	0.004	1.28 ± 0.06	1.35 ± 0.14	0.34
ΔD (×10^−3^ mm^2^/s)	0.005 ± 0.10	0.04 ± 0.09	−0.02 ± 0.10	<0.001	0.03 ± 0.10	−0.04 ± 0.12	0.04	0.05 ± 0.09	−0.03 ± 0.09	<0.001	0.12 [0.08, 0.15]	0.09 [−0.05, 0.13]	0.40
D*c (×10^−3^ mm^2^/s)	10.90 [7.12, 17.20]	11.30 [7.78, 17.50]	10.60 [6.80, 16.70]	0.17	10.40 [7.43, 16.10]	7.17 [5.53, 11.70]	0.04	12.80 [8.04, 17.70]	10.80 [7.26, 16.80]	0.97	27.90 [23.90, 32.00]	12.70 [8.41, 21.50]	0.38
D*m (×10^−3^ mm^2^/s)	9.79 [6.99, 15.40]	10.10 [7.35, 16.80]	9.61 [6.96, 15.00]	0.33	9.20 [6.76, 15.20]	6.78 [4.68, 13.20]	0.26	11.70 [7.97, 18.50]	9.87 [7.65, 14.60]	0.45	14.40 ± 0.41	12.80 ± 7.33	0.38
ΔD* (×10^−3^ mm^2^/s)	0.24 [−1.67, 2.59]	0.47 [−1.60, 3.07]	0.19 [−1.62, 1.64]	0.53	0.90 [−0.98, 3.05]	0.34 [−0.47, 0.82]	0.54	−0.14 [−2.67, 1.94]	−0.02 [−1.67, 3.29]	0.26	13.50 [9.36, 17.70]	0.43 [−2.68, 7.26]	0.45
fc (%)	27.60 [24.00, 31.20]	27.50 [24.50, 31.30]	27.60 [23.80, 30.80]	0.63	28.40 ± 4.48	26.40 ± 4.55	0.08	25.90 [23.20, 31.10]	28.00 [24.20, 31.80]	0.22	33.00 [28.80, 37.20]	25.90 [23.50, 28.70]	0.57
fm (%)	28.60 ± 5.49	28.90 ± 5.53	28.40 ± 5.47	0.56	29.30 ± 5.88	27.10 ± 6.27	0.15	28.00 ± 4.38	28.80 ± 5.28	0.42	33.20 ± 15.20	28.00 ± 5.29	0.72
Δf (%)	−0.55 [−2.65, 2.12]	−0.45 [−2.79, 2.16]	−0.62 [−2.61, 1.76]	0.88	−0.44 [−2.75, 2.14]	0.47 [−2.78, 1.79]	0.83	−0.50 [−2.85, 2.36]	−0.68 [−2.65, 2.17]	0.64	−0.18 [−1.32, 0.96]	−0.92 [−1.65, 0.41]	0.62
T1c (ms)	1410.00 ± 92.40	1370.00 ± 81.90	1430.00 ± 92.90	<0.001	1380.00 ± 85.80	1420.00 ± 117.00	0.12	1370.00 ± 76.80	1440.00 ± 84.70	<0.001	1370.00 ± 85.00	1400.00 ± 94.50	0.67
T1m (ms)	1540.00 ± 107.00	1540.00 ± 114.00	1540.00 ± 101.00	0.84	1560.00 ± 118.00	1550.00 ± 123.00	0.58	1500.00 ± 95.70	1540.00 ± 92.00	0.03	1400.00 ± 79.80	1520.00 ± 112.00	0.25
ΔT1 (ms)	−131.00 ± 92.10	−161.00 ± 96.10	−108.00 ± 81.80	<0.001	−182.00 ± 94.80	−124.00 ± 89.30	0.01	−135.00 ± 90.20	−102.00 ± 81.90	0.05	−36.10 [−37.90, −34.30]	−95.50 [−188.00, −59.40]	<0.001
Flux (mL/s)	3.77 [2.63, 4.79]	4.00 [2.83, 6.00]	3.58 [2.47, 4.51]	0.01	3.95 [2.79, 5.85]	3.28 [2.60, 4.00]	0.002	4.48 [2.92, 6.33]	3.69 [2.45, 4.65]	0.03	3.60 ± 0.81	3.57 ± 1.33	0.97
Stroke volume (mL)	3.18 [2.26, 4.35]	3.50 [2.45, 4.96]	2.92 [2.10, 4.21]	0.01	3.54 [2.45, 4.93]	2.50 [2.23, 3.30]	<0.001	3.49 [2.52, 5.74]	3.07 [2.08, 4.26]	0.05	2.67 ± 0.54	3.44 ± 1.51	0.23
Velocity (cm/s)	8.99 [5.26, 11.70]	9.90 [6.49, 13.30]	7.67 [4.80, 10.30]	<0.001	10.20 [7.24, 13.40]	9.18 [7.21, 11.30]	0.05	9.21 [5.65, 13.10]	6.76 [4.31, 9.46]	0.004	11.20 ± 6.76	10.20 ± 4.26	0.86
RBFc (mL/100 g/min)	92.60 [72.60, 121.00]	102.00 [81.60, 125.00]	87.30 [60.00, 118.00]	0.004	107.00 ± 30.50	96.30 ± 36.90	0.24	93.40 [76.10, 125.00]	85.30 [56.60, 105.00]	0.02	99.30 ± 6.75	103.00 ± 28.30	0.67
RBFm (mL/100 g/min)	19.00 [14.20, 23.90]	20.10 [16.00, 23.00]	17.00 [12.90, 24.30]	0.054	19.80 [14.90, 22.90]	20.80 [13.60, 27.50]	0.59	20.60 [17.80, 23.30]	16.50 [12.70, 26.60]	0.45	21.60 ± 2.01	17.90 ± 5.87	0.14
T2*c (ms)	42.60 [37.00, 47.40]	42.60 [37.50, 46.90]	42.40 [36.20, 48.10]	0.90	42.50 ± 5.66	40.80 ± 8.73	0.38	42.80 [35.90, 46.40]	43.10 [35.60, 47.60]	0.81	44.10 ± 3.04	43.40 ± 5.43	0.82
T2*m (ms)	36.20 [30.70, 42.20]	37.90 [31.70, 43.00]	35.10 [30.50, 41.40]	0.11	41.60 [34.30, 45.20]	37.50 [32.20, 42.50]	0.23	34.00 ± 5.67	36.20 ± 8.26	0.09	38.80 ± 4.58	32.20 ± 5.58	0.26
ΔT2* (ms)	4.92 ± 7.66	4.46 ± 7.97	5.28 ± 7.41	0.64	2.60 ± 8.07	2.91 ± 7.82	0.87	7.51 [1.44, 13.20]	5.05 [−0.94, 10.00]	0.07	5.29 ± 7.62	11.20 ± 6.90	0.46

Note: Continuous variables are reported as medians with interquartile ranges or means ± standard deviations, depending on data distribution. Categorical variables are summarized as frequency counts (*n*) and percentages (%). *p*-values were adjusted for multiple comparisons using the false discovery rate (FDR) method. c: cortex; m: medulla; Δ: difference (cortex—medulla); RBF: renal blood flow.

**Table 3:** Univariate and multivariate logistic regression analysis for renal interstitial fibrosis (RIF) evaluation.

Predictor Variable	Unadjusted OR	Adjusted OR
	(95% CI, *p* Value)	(95% CI, *p* Value)
Age (per 1-SD = 13.48 years)	0.76 (0.58–1.14, *p* = 0.16)	
Sex		
Female	ref	
Male	1.73 (0.89–3.37, *p* = 0.11)	
Disease		
Acute kidney injury	ref	
Diabetic kidney disease	4.74 (2.34–9.58, *p* < 0.001)	
IgA nephropathy	6.60 (1.22–35.60, *p* = 0.03)	
Body mass index (per 1-SD = 3.99 kg/m^2^)	0.85 (0.60–1.22, *p* = 0.34)	
Mean arterial pressure (per 1-SD = 13.20 mmHg)	1.00 (0.77–1.48, *p* = 0.74)	
Serum creatinine (per 1-SD = 218.92 μmol/L)	1.00 (1.00–1.00, *p* = 0.64)	
Estimated glomerular filtration rate (per 1-SD = 17.55 mL/min/1.73 m^2^)	0.84 (0.59–1.19, *p* = 0.14)	
Hemoglobin (per 1-SD = 23.37 g/L)	1.26 (1.00–1.59, *p* = 0.17)	2.50 (1.26–4.86, *p* = 0.005)
Albumin (per 1-SD = 6.54 g/L)	0.77 (0.54–1.00, *p* = 0.08)	
C-reactive protein (per 1-SD = 15.19 mg/L)	1.00 (0.74–1.35, *p* = 0.79)	
D-dimer (per 1-SD = 0.69 mg/L)	0.72 (0.46–1.11, *p* = 0.13)	
BUN (per 1-SD = 6.58 mmol/L)	1.38 (1.00–1.87, *p* = 0.05)	1.99 (1.00–4.12, *p* = 0.04)
UTP (per 1-SD = 4.65 g/24 h)	2.72 (1.62–4.46, *p* < 0.001)	2.72 (1.20–6.18, *p* = 0.02)
Kidney length (per 1-SD = 1.14 cm)	0.98 (0.71–1.35, *p* = 0.88)	
Height-adjusted total kidney volume (per 1-SD = 24.13 mL/m)	0.78 (0.61–1.00, *p* = 0.18)	
Cortico-medullary boundary		
Distinct boundary	ref	
Blurred boundary	1.83 (0.51–6.51, *p* = 0.35)	
Obliterated boundary	4.00 (1.06–15.08, *p* = 0.04)	
Dc (per 1-SD = 0.13 × 10^−3^ mm^2^/s)	0.48 (0.34–0.69, *p* < 0.001)	
Dm (per 1-SD = 0.13 × 10^−3^ mm^2^/s)	0.91 (0.67–1.26, *p* = 0.57)	
ΔD (per 1-SD = 0.10 × 10^−3^ mm^2^/s)	1.00 (1.00–1.00, *p* < 0.001)	1.00 (1.00–1.01, *p* = 0.004)
D*c (per 1-SD = 9.88 × 10^−3^ mm^2^/s)	0.67 (0.44–1.00, *p* = 0.04)	
D*m (per 1-SD = 10.95 × 10^−3^ mm^2^/s)	0.72 (0.45–1.12, *p* = 0.14)	0.36 (0.15–0.72, *p* = 0.008)
ΔD* (per 1-SD = 8.94 × 10^−3^ mm^2^/s)	0.91 (0.63–1.30, *p* = 0.55)	
fc (per 1-SD = 4.72%)	0.91 (0.67–1.26, *p* = 0.57)	
fm (per 1-SD = 5.49%)	0.85 (0.60–1.11, *p* = 0.25)	
Δf (per 1-SD = 4.03%)	1.13 (0.85–1.58, *p* = 0.40)	
T1c (per 1-SD = 92.37 ms)	2.51 (1.00–2.51, *p* = 0.003)	2.51 (1.00–6.23, *p* = 0.02)
T1m (per 1-SD = 106.60 ms)	1.00 (1.00–1.00, *p* = 0.93)	
ΔT1 (per 1-SD = 92.09 ms)	2.50 (1.00–2.50, *p* = 0.004)	
Flux (per 1-SD = 1.97 mL/s)	0.51 (0.34–0.74, *p* < 0.001)	
Stroke volume (per 1-SD = 1.66 mL)	0.49 (0.33–0.71, *p* < 0.001)	0.22 (0.06–0.75, *p* = 0.02)
Velocity (per 1-SD = 4.55 cm/s)	0.38 (0.25–0.59, *p* < 0.001)	0.38 (0.18–0.83, *p* = 0.01)
RBFc (per 1-SD = 34.40 mL/100 g/min)	0.71 (0.50–1.00, *p* = 0.02)	
RBFm (per 1-SD = 7.65 mL/100 g/min)	1.08 (0.79–1.45, *p* = 0.49)	
T2*c (per 1-SD = 7.28 ms)	0.86 (0.59–1.16, *p* = 0.30)	
T2*m (per 1-SD = 7.58 ms)	0.63 (0.45–0.93, *p* = 0.02)	0.45 (0.27–0.79, *p* = 0.006)

Note: OR: odds ratio; CI: confidence interval; c: cortex; m: medulla; Δ: difference (cortex—medulla); RBF: renal blood flow; SD: standard deviation; UTP: urinary total protein; BUN: blood urea nitrogen. All continuous variables were rescaled and are reported per 1 standard deviation. Categorical variables (sex, disease, and cortico-medullary boundary) are reported as crude odds ratios. Raw (unadjusted) *p*-values are presented.

**Table 4:** Measures of variable importance for the random forest model.

Variable	Gini Decrease	Mean Minimum Depth	Number of Nodes	Accuracy Decrease	Number of Trees	Times as Root
UTP	16.217	1.375	1972	0.055	496	181
ΔD	13.505	1.554	1914	0.025	498	78
Dc	13.422	1.833	1899	0.034	498	54
Velocity	12.148	1.840	1687	0.022	486	116
T1c	8.994	2.433	1762	0.014	496	9
ΔT1	8.567	2.548	1642	0.010	488	9
Disease	3.834	3.729	418	0.012	313	53

Note: UTP: urine total protein; c: cortex; m: medulla; Δ: difference (cortex—medulla).

**Table 5:** Classification performance of the RF model and LASSO-based models in the internal held-out test set.

Models	AUC	ACC	SEN	SPE	PPV	NPV
RF Model	0.890 (0.816, 0.964)	0.842 (0.839, 0.846)	0.913 (0.832, 0.994)	0.733 (0.575, 0.892)	0.840 (0.738, 0.942)	0.846 (0.707, 0.985)
Combined LASSO LR Model	0.730 (0.613, 0.848)	0.763 (0.758, 0.768)	0.913 (0.832, 0.994)	0.533 (0.355, 0.712)	0.750 (0.637, 0.863)	0.800 (0.625, 0.975)
MR LASSO LR Model	0.632 (0.494, 0.770)	0.737 (0.732, 0.742)	0.957 (0.898, 1.000)	0.400 (0.225, 0.575)	0.710 (0.597, 0.823)	0.857 (0.674, 1.040)
Clinical LASSO LR Model	0.625 (0.493, 0.758)	0.658 (0.652, 0.664)	0.804 (0.690, 0.919)	0.433 (0.256, 0.611)	0.685 (0.561, 0.809)	0.591 (0.385, 0.796)

Note: AUC: area under the receiver operating characteristic curve; ACC: accuracy; SEN: sensitivity; SPE: specificity; PPV: positive predictive value; NPV: negative predictive value.

## Data Availability

The data presented in this study are only available on reasonable request from the corresponding author due to ethical and privacy restrictions. The data are not publicly available as they contain information that could compromise the privacy of the research participants.

## Supplementary Materials

The following supporting information can be downloaded at: https://www.mdpi.com/article/10.3390/bioengineering13060704/s1, Figure S1: Region-of-interest placements in the bilateral kidneys; Table S1: The MRI acquisition parameters; Table S2: Inter-observer reproducibility for MRI parameters; Table S3: Diagnostic performance of different classification methods for renal interstitial fibrosis (RIF) evaluation; Figure S2: Comparison of model performance in the training and internal held-out test sets.

## Abbreviations

The following abbreviations are used in this manuscript:

RIF	Renal interstitial fibrosis
IVIM	Intravoxel incoherent motion
ASL	Arterial spin labeling
BOLD	Blood oxygen level-dependent
ROC	Receiver operating characteristic
AUC	Area under the curve
UTP	Urine total protein
BUN	Blood urea nitrogen
OR	Odds ratio
