# Supplementary material for: Machine Learning with Multiparametric MRI and Clinical Biomarkers for Noninvasive Renal Interstitial Fibrosis Staging
Source: Bioengineering (Basel). 2026 Jun 19;13(6):704. doi: 10.3390/bioengineering13060704 (PMC13295375; doi:10.3390/bioengineering13060704)
Supplement: Supplementary file 1 [file bioengineering-13-00704-s001.zip › bioengineering-4295087-supplementary.pdf]

Supplementary Materials

Figure S1: Regions of interest placements in the bilateral kidneys. On coronal renal hilum section, regions of interest were manually positioned at the upper, middle, and lower poles of bilateral kidneys on multiparametric maps: (a) intravoxel incoherent motion (IVIM)-derived pure diffusion coefficient (D), perfusion fraction (f), and pseudo-diffusion coefficient (D*) maps, (b) T1 mapping-derived T1 map, (c) arterial spin labeling (ASL)-derived renal blood flow (RBF) map, and (d) blood oxygen level-dependent (BOLD)-derived T2* map. Regions of interest were deliberately positioned to avoid biopsy tracts, the collecting system, vessels, and artifacts.

Figure S2: Comparison of model performance in the training and test Sets. (a) Training set performance. The scatter plot displays AUC values for 17 classification methods. (b) Independent test set performance. A mild performance decline is observed compared to the training set. The random forest model maintained superior discrimination (AUC=0.88). UTP: urine total protein, BUN: blood urea nitrogen, RBF: renal blood flow.

Table S1 The MRI acquisition parameters

Table S2 Inter-observer reproducibility for MRI parameters

Table S3: Diagnostic performance of different classification methods for renal interstitial fibrosis (RIF) evaluation.

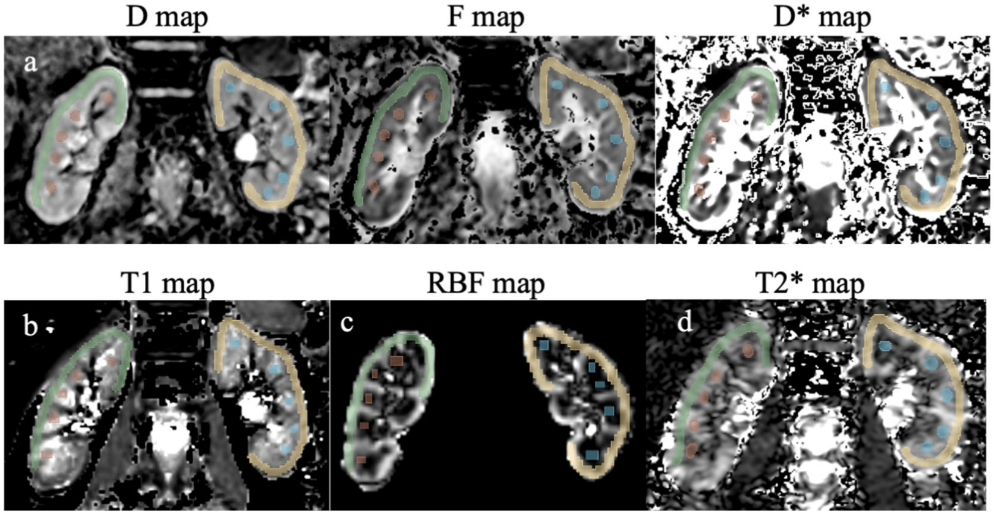

Figure S1: Regions of interest placements in the bilateral kidneys. On coronal renal hilum section, regions of interest were manually positioned at the upper, middle, and lower poles of bilateral kidneys on multiparametric maps: (a) intravoxel incoherent motion (IVIM)-derived pure diffusion coefficient (D), perfusion fraction (f), and pseudo-diffusion coefficient (D*) maps, (b) T1 mapping-derived T1 map, (c) arterial spin labeling (ASL)-derived renal blood flow (RBF) map, and (d) blood oxygen level-dependent (BOLD)-derived T2* map. Regions of interest were deliberately positioned to avoid biopsy tracts, the collecting system, vessels, and artifacts.

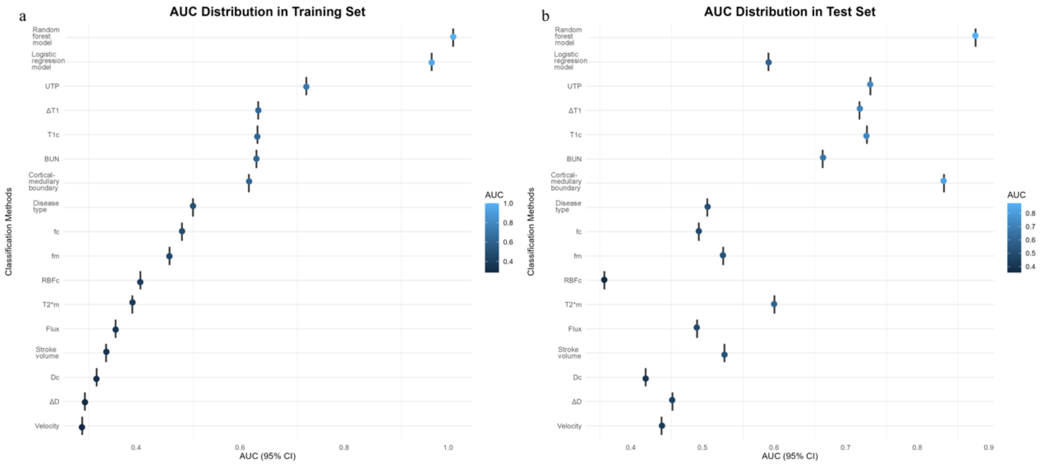

Figure S2: Comparison of model performance in the training and test Sets. (a) Training set performance. The scatter plot displays AUC values for 17 classification methods. (b) Independent test set performance. A mild performance decline is observed compared to the training set. The random forest model maintained superior discrimination (AUC=0.88). UTP: urine total protein, BUN: blood urea nitrogen, RBF: renal blood flow.

Table S1 The MRI acquisition parameters

	Dixon T1-WI	T2-WI	IVIM	T1 mapping	ASL	BOLD	Phase-contrast imaging
Sequence type	FFE	TSE	EPI	IR	pCASL	EPI	TFE
Imaging plane	Transverse	Coronal	Coronal	Coronal	Coronal	Coronal	Sagittal
Flip angle (°)	10	90	90	6	90	30	25
TE (ms)	1.2 / 2.4	74.1	78.9	1.92	19	16	4.7
TR (ms)	3.7	3000	1300	4.15	4550	435	8.0
Slice thickness (mm)	4	4	8	8	8	8	5
Bandwidth (kHz)	1.3	1.7	2.3	0.38	2	2	0.224
Matrix	288 × 191	256×238	120×117	180×180	120×120	144×118	128×256
Field of view (cm^2)	40×32	36×36	36×36	36×36	36×36	36×36	15×30
Scan time	13 s	1 min	5 min 42 s	20s	4 min 42 s	17s	36s

T1-WI: T1-weighted imaging, T2-WI: T2-weighted imaging, IVIM: intravoxel incoherent motion, ASL: arterial spin labeling, BOLD: blood-oxygen-level-dependent, FFE: fast field echo, TSE: turbo spin echo, EPI: echo planar imaging, IR: inversion recovery, pCASL: pseudocontinuous arterial spin labeling, TFE: turbo field echo, TE: echo time, TR: repetition time.

Table S2 Inter-observer reproducibility for MRI parameters

	Intraclass correlation coefficient (95% CI)
Dc	0.78 (0.72-0.82)
Dm	0.82 (0.77-0.86)
fc	0.73 (0.66-0.78)
fm	0.75 (0.69-0.80)
D*c	0.78 (0.73-0.83)
D*m	0.71 (0.64-0.78)
T1c	0.84 (0.79-0.87)
T1m	0.72 (0.65-0.78)
Flux	0.84 (0.80-0.88)
Stroke volume	0.82 (0.78-0.86)
Velocity	0.85 (0.81-0.88)
RBFc	0.70 (0.63-0.76)
RBFm	0.69 (0.62-0.76)
T2*c	0.85 (0.81-0.88)
T2*m	0.65 (0.56-0.71)

CI: confidence interval, c: cortex, m: medulla, Δ: difference (cortex - medulla), RBF: renal blood flow.

Table S3 Diagnostic performance of different classification methods for renal interstitial fibrosis (RIF) evaluation

	AUC	Accuracy	Sensitivity	Specificity	PPV	NPV	PLR	NLR
**Training Set**								
Random forest model	1.00 (1.00, 1.00)	1.00 (1.00, 1.00)	1.00 (1.00, 1.00)	1.00 (1.00, 1.00)	1.00 (1.00, 1.00)	1.00 (1.00, 1.00)	Inf (NaN, Inf)	0.00 (0.00, NaN)
Logistic regression model	0.96 (0.93, 0.99)	0.92 (0.91, 0.92)	0.91 (0.85, 0.98)	0.92 (0.85, 0.98)	0.93 (0.87, 0.98)	0.90 (0.84, 0.97)	10.98 (5.09, 23.68)	0.09 (0.05, 0.19)
UTP	0.72 (0.64, 0.80)	0.70 (0.70, 0.70)	0.56 (0.45, 0.67)	0.86 (0.78, 0.94)	0.82 (0.72, 0.92)	0.63 (0.54, 0.73)	4.04 (2.20, 7.41)	0.51 (0.39, 0.66)
Disease type	0.69 (0.61, 0.76)	0.69 (0.69, 0.69)	0.76 (0.66, 0.85)	0.61 (0.50, 0.72)	0.69 (0.59, 0.79)	0.69 (0.57, 0.80)	1.94 (1.42, 2.66)	0.40 (0.26, 0.61)
ΔT1	0.63 (0.54, 0.71)	0.62 (0.62, 0.63)	0.90 (0.84, 0.97)	0.31 (0.20, 0.41)	0.60 (0.51, 0.68)	0.73 (0.58, 0.89)	1.30 (1.10, 1.54)	0.32 (0.15, 0.67)
T1c	0.62 (0.54, 0.71)	0.60 (0.60, 0.61)	0.56 (0.45, 0.67)	0.65 (0.54, 0.76)	0.65 (0.54, 0.76)	0.57 (0.46, 0.67)	1.62 (1.12, 2.34)	0.67 (0.50, 0.91)
BUN	0.62 (0.53, 0.71)	0.62 (0.62, 0.63)	0.46 (0.36, 0.57)	0.81 (0.71, 0.90)	0.73 (0.61, 0.85)	0.57 (0.47, 0.67)	2.38 (1.41, 4.03)	0.67 (0.53, 0.84)
Cortico-medullary boundary	0.61 (0.53, 0.69)	0.58 (0.58, 0.59)	0.44 (0.33, 0.55)	0.75 (0.65, 0.85)	0.67 (0.54, 0.79)	0.54 (0.44, 0.64)	1.76 (1.10, 2.81)	0.75 (0.59, 0.95)
fc	0.48 (0.39, 0.57)	0.53 (0.53, 0.54)	0.48 (0.37, 0.58)	0.60 (0.48, 0.71)	0.57 (0.46, 0.69)	0.50 (0.39, 0.61)	1.18 (0.82, 1.70)	0.88 (0.66, 1.16)
fm	0.46 (0.36, 0.55)	0.51 (0.51, 0.52)	0.48 (0.37, 0.58)	0.56 (0.44, 0.67)	0.55 (0.43, 0.67)	0.48 (0.37, 0.59)	1.07 (0.76, 1.51)	0.94 (0.71, 1.26)
RBFc	0.40 (0.31, 0.49)	0.48 (0.48, 0.48)	0.15 (0.07, 0.22)	0.86 (0.78, 0.94)	0.55 (0.34, 0.75)	0.47 (0.39, 0.56)	1.05 (0.48, 2.29)	0.99 (0.87, 1.13)
T2*m	0.38 (0.29, 0.47)	0.49 (0.49, 0.50)	0.17 (0.09, 0.25)	0.86 (0.78, 0.94)	0.58 (0.39, 0.78)	0.48 (0.39, 0.56)	1.23 (0.58, 2.60)	0.96 (0.84, 1.10)
Flux	0.35 (0.26, 0.44)	0.47 (0.46, 0.47)	1.00 (1.00, 1.00)	0.00 (0.00, 0.00)	NaN (NaN, NaN)	0.47 (0.39, 0.55)	1.00 (NaN, NaN)	NaN (NaN, NaN)
Stroke volume	0.33 (0.25, 0.42)	0.47 (0.46, 0.47)	1.00 (1.00, 1.00)	0.00 (0.00, 0.00)	NaN (NaN, NaN)	0.47 (0.39, 0.55)	1.00 (NaN, NaN)	NaN (NaN, NaN)
Dc	0.32 (0.23, 0.40)	0.47 (0.46, 0.47)	1.00 (1.00, 1.00)	0.00 (0.00, 0.00)	NaN (NaN, NaN)	0.47 (0.39, 0.55)	1.00 (NaN, NaN)	NaN (NaN, NaN)
ΔD	0.29 (0.21, 0.37)	0.47 (0.46, 0.47)	1.00 (1.00, 1.00)	0.00 (0.00, 0.00)	NaN (NaN, NaN)	0.47 (0.39, 0.55)	1.00 (NaN, NaN)	NaN (NaN, NaN)
Velocity	0.29 (0.21, 0.37)	0.47 (0.46, 0.47)	1.00 (1.00, 1.00)	0.00 (0.00, 0.00)	NaN (NaN, NaN)	0.47 (0.39, 0.55)	1.00 (NaN, NaN)	NaN (NaN, NaN)
**Internally Hold-out Test Set**								
Random forest model	0.88 (0.80, 0.95)	0.83 (0.83, 0.84)	0.90 (0.81, 0.98)	0.73 (0.58, 0.89)	0.84 (0.74, 0.94)	0.82 (0.67, 0.96)	3.36 (1.84, 6.13)	0.14 (0.06, 0.34)
Cortico-medullary boundary	0.80 (0.73, 0.81)	0.80 (0.79, 0.80)	0.71 (0.58, 0.84)	0.93 (0.84, 1.00)	0.94 (0.87, 1.02)	0.67 (0.52, 0.81)	10.63 (2.75, 41.03)	0.31 (0.20, 0.49)
Disease type	0.78 (0.70, 0.87)	0.77 (0.77, 0.77)	0.92 (0.84, 1.00)	0.53 (0.36, 0.71)	0.76 (0.65, 0.87)	0.80 (0.63, 0.98)	1.96 (1.33, 2.91)	0.16 (0.06, 0.42)

UTP	0.73 (0.61, 0.84)	0.74 (0.74, 0.75)	0.71 (0.58, 0.84)	0.80 (0.66, 0.94)	0.85 (0.74, 0.96)	0.63 (0.48, 0.79)	3.54 (1.69, 7.41)	0.37 (0.23, 0.59)
T1c	0.72 (0.61, 0.84)	0.69 (0.69, 0.70)	0.56 (0.42, 0.70)	0.90 (0.79, 1.00)	0.90 (0.79, 1.01)	0.56 (0.42, 0.70)	5.63 (1.87, 16.94)	0.49 (0.35, 0.68)
ΔT1	0.71 (0.60, 0.83)	0.68 (0.67, 0.69)	0.71 (0.58, 0.84)	0.63 (0.46, 0.81)	0.76 (0.63, 0.88)	0.58 (0.41, 0.74)	1.93 (1.17, 3.20)	0.46 (0.27, 0.77)
BUN	0.66 (0.54, 0.79)	0.69 (0.69, 0.70)	0.79 (0.68, 0.91)	0.53 (0.36, 0.71)	0.73 (0.61, 0.85)	0.62 (0.43, 0.80)	1.70 (1.13, 2.55)	0.39 (0.21, 0.75)
T2*m	0.59 (0.47, 0.72)	0.64 (0.64, 0.65)	0.71 (0.58, 0.84)	0.53 (0.36, 0.71)	0.71 (0.58, 0.84)	0.53 (0.36, 0.71)	1.52 (0.99, 2.32)	0.55 (0.31, 0.95)
Logistic regression model	0.59 (0.45, 0.72)	0.69 (0.69, 0.70)	0.94 (0.87, 1.00)	0.30 (0.14, 0.46)	0.68 (0.57, 0.79)	0.75 (0.51, 1.00)	1.34 (1.05, 1.71)	0.21 (0.06, 0.71)
Stroke volume	0.52 (0.38, 0.67)	0.68 (0.67, 0.69)	0.92 (0.84, 1.00)	0.30 (0.14, 0.46)	0.68 (0.56, 0.79)	0.69 (0.44, 0.94)	1.31 (1.02, 1.68)	0.28 (0.09, 0.82)
fm	0.52 (0.39, 0.66)	0.56 (0.56, 0.57)	0.52 (0.38, 0.66)	0.63 (0.46, 0.81)	0.69 (0.54, 0.85)	0.45 (0.30, 0.60)	1.42 (0.83, 2.45)	0.76 (0.51, 1.13)
fc	0.49 (0.35, 0.62)	0.56 (0.56, 0.57)	0.63 (0.49, 0.76)	0.47 (0.29, 0.65)	0.65 (0.52, 0.79)	0.44 (0.27, 0.61)	1.17 (0.79, 1.75)	0.80 (0.47, 1.36)
Flux	0.49 (0.34, 0.63)	0.64 (0.64, 0.65)	0.85 (0.75, 0.95)	0.30 (0.14, 0.46)	0.66 (0.54, 0.78)	0.56 (0.32, 0.81)	1.22 (0.94, 1.59)	0.49 (0.20, 1.17)
ΔD	0.45 (0.32, 0.58)	0.51 (0.51, 0.52)	0.42 (0.28, 0.56)	0.67 (0.50, 0.84)	0.67 (0.50, 0.84)	0.42 (0.28, 0.56)	1.25 (0.68, 2.29)	0.88 (0.62, 1.24)
Velocity	0.44 (0.30, 0.57)	0.62 (0.61, 0.62)	0.92 (0.84, 1.00)	0.13 (0.01, 0.26)	0.63 (0.52, 0.74)	0.50 (0.15, 0.85)	1.06 (0.90, 1.25)	0.63 (0.17, 2.31)
Dc	0.41 (0.28, 0.55)	0.58 (0.57, 0.58)	0.79 (0.68, 0.91)	0.23 (0.08, 0.39)	0.62 (0.50, 0.75)	0.41 (0.18, 0.65)	1.03 (0.81, 1.32)	0.89 (0.38, 2.09)
RBFc	0.36 (0.23, 0.48)	0.39 (0.38, 0.39)	1.00 (1.00, 1.00)	0.00 (0.00, 0.00)	NaN (NaN, NaN)	0.39 (0.28, 0.49)	1.00 (NaN, NaN)	NaN (NaN, NaN)

AUC: area under the receiver operating characteristic curve, PPV: positive predictive value, NPV: negative predictive value, PLR: positive likelihood ratio, NLR: negative likelihood ratio, UTP: urine total pro-tein, BUN: blood urea nitrogen, c: cortex, m: medulla, Δ: difference (cortex - medulla), RBF: renal blood flow.
